# 我如何治疗老年急性髓系白血病

**DOI:** 10.3760/cma.j.issn.0253-2727.2021.09.003

**Published:** 2021-09

**Authors:** 洁 金

**Affiliations:** 浙江大学医学院附属第一医院血液科，浙江省血液肿瘤（诊治）重点实验室，浙江大学癌症中心，浙江省血液病临床医学研究中心，杭州 310003 Department of Hematology, the First Affiliated Hospital of Zhejiang University, College of Medicine; Zhejiang Province Key Laboratory of Hematology Oncology Diagnosis and Treatment; Cancer Center of Zhejiang University; Zhejiang Provincial Clinical Research Center For Hematologic Diseases, Hangzhou 310003, China

急性髓系白血病（AML）是一组造血组织来源的恶性克隆性疾病，其特征为异常原始细胞的堆积以及正常血细胞的生成及分化障碍。老年AML患者具有异质性强、一般情况差、合并症多、不良预后因素多等特点，其治疗给血液科临床医师带来挑战。近年来随着多种新药的出现和应用，AML的治疗取得了快速进展。本文通过介绍1例老年AML患者的诊治经过，总结本中心老年AML的个体化治疗及新药应用的经验，供同行借鉴。

一、典型病例

男，86岁，因“双下肢水肿半个月，发现血三系减少1 d”就诊。血常规：WBC 0.9×10^9^/L，RBC 1.24×10^12^/L，HGB 46 g/L，PLT 25×10^9^/L。骨髓象：一类原始细胞比例增高（占36％），过氧化物酶染色阳性率2％，形态学考虑M_0_可能。免疫分型原始髓系细胞占28.34％，表达CD117、CD34、CD13、HLA-DR、CD38、CD123，提示AML。43种白血病融合基因检查报告阴性，33个白血病基因突变筛查提示BCORL1 36.40％，ETV6 17.50％，FLT3-ITD 8.70％，TET2 98.94％，染色体核型为46,XY。患者入院肺部CT提示：右肺上中叶及左肺上叶支气管扩张症伴多发感染。头颅MRI提示：左侧侧脑室旁急性腔梗灶。最终诊断为AML（M_0_，ELN2017预后分层为中危组），ECOG评分2分。对于该患者，我们按照Ferrara评判是否耐受强烈化疗的标准判断该患者不适合强烈化疗，患者年龄>75岁，同时还合并肺部感染和急性脑梗死，化疗风险极大，但患者及家属有强烈的治疗意愿。因此，我们为该患者选择了维奈克拉（Venetoclax）联合阿扎胞苷的治疗方案。具体治疗方法如下：维奈克拉100 mg第1天、200 mg第2天、300 mg第3天、400 mg第4～28天，阿扎胞苷75 mg/m^2^第1～7天，治疗期间予以红细胞、血小板输注支持。28 d（1个疗程）后评估患者达到完全缓解（CR），微小残留病（MRD）为0.070％。2个疗程后MRD<0.01％，后继续维奈克拉+阿扎胞苷方案减量维持治疗。整个治疗过程中，患者耐受性好，生活质量佳。随访至今10个月，为持续缓解状态。

二、老年AML的现状及特点

老年患者一般指年龄≥65岁的患者，AML的中位发病年龄为68～70岁，意味着一半以上的患者为老年患者。老年患者中治疗相关AML以及继发性AML占比高（20％），体能状态差，高血压、糖尿病等合并症多，有患者伴有肝肾功能损害，多数不能耐受强化化疗，使得治疗选择局限，治疗难度大。

细胞遗传学方面，老年AML患者更倾向于携带预后不良的核型，如5号、7号染色体的丢失及异常，inv（3q），复杂核型，而携带预后良好相关核型改变，如t（16;16）、t（8;21）、t（15;17）的患者比例较年轻患者显著降低[1]–[2]。

分子遗传学方面,老年患者携带疾病驱动基因突变数目随着年龄增大而显著增多。在<40岁的患者中仅有20％的患者携带3个以上突变的疾病驱动基因，而在≥60岁的患者中这一比例高达50％[3]。与年轻患者相比，老年患者携带预后不良相关基因突变（如TP53突变）比例增高；而携带预后良好相关基因突变（如CEBPA双突变）比例则降低[4]。并且老年患者更倾向于携带某一群特定基因的突变，如TET2、RUNX1、SRSF2、ASXL1、SF3B1，这一群基因在近年来被定义为克隆造血相关基因（Clonal Hematopoiesis）[3],[5],提示可能与白血病发生发展过程中恶性克隆的逐步演变相关。

具有以上不良预后因素的老年AML患者，预计强烈化疗效果不理想。但并不是所有老年患者都不适合强烈化疗，将适合强烈化疗者定义为fit患者，不适合化疗者定义为unfit患者，目前多采用Ferrara的标准判断患者是否为unfit患者。满足以下至少一项标准表明为unfit患者：①高龄：>75岁。②严重的心脏合并症：充血性心力衰竭或既往有射血分数（EF）≤50％的心肌病史。③严重的肺部合并症：既往有肺部疾病史，肺一氧化碳弥散量（DLCO）≤65％或第一秒用力呼气容积（FEV1）≤65％，或静止时仍存在呼吸困难或需要吸氧，或任何胸膜肿瘤或未得到控制的肺部肿瘤。④严重的肾脏合并症：>60岁且正接受透析治疗，或未得到控制的肾脏肿瘤。⑤严重的肝脏合并症：Child B或C级的肝硬化，或年龄≥60岁且伴有导致转氨酶大幅升高（>正常值的3倍）的肝病，或任何胆管癌，或未得到控制的肝癌或急性病毒性肝炎。⑥对抗感染治疗无效的活动性感染。⑦存在认知障碍：目前伴有需要在精神病院或管制机构住院治疗，或加强门诊管理的精神类疾病，或当前存在不受照顾者控制的依赖性认知状态（由专科医生确诊）。⑧体能状态差（ECOG评分）：与白血病无关的ECOG体能状态评分≥3。⑨医生判断的不适合进行化疗的其他合并症。

三、老年AML的治疗

数据分析得出，仅有35％～50％的老年患者在确诊疾病的3个月内接受治疗，并且随着确诊年龄的增大接受治疗的患者比例逐渐减低[6]。年龄大于70岁的患者，许多仅接受姑息治疗和临终关怀。MD Anderson中心数据分析提示，老年AML的强烈化疗缓解率为40％～50％，1～2个月内的早期病死率高达26％～36％，中位生存仅4～6个月，1年总生存（OS）率低于30％，与年轻患者缓解率70％～80％、长期生存率40％～50％相比差距较大[7]。

老年AML患者治疗方案的选择需要根据患者一般情况和疾病的生物学特征进行综合性、个体化评估。这些患者在诊断和治疗过程中可能出现严重的骨髓抑制和血细胞减少。治疗过程中预防性的抗生素使用和及时的支持治疗（血液产品）、重症感染的预判，并在需要时及时使用重症监护室护理均非常重要，否则患者病死率极高。鉴于老年AML患者强烈化疗缓解率低、治疗相关死亡率高，临床医师不得不思考低强度的化疗方案，以及如何选择适合治疗的患者。目前在老年患者中的治疗突破，主要为小分子靶向药物单用或联合去甲基化药物（HMA）的应用。

1. 维奈克拉：维奈克拉是一种选择性的BCL-2抑制剂，可特异性地结合于抗凋亡蛋白BCL-2的BH3结构域，从而解除BCL-2对促凋亡蛋白的抑制作用，最终促进白血病细胞凋亡[8]。鉴于其在AML中优越的疗效，2018年11月21日FDA加急批准了维奈克拉与HMA或低剂量阿糖胞苷（LDAC）联合应用于>75岁的老年初治AML患者或不适合强化疗患者，该药于2020年12月在国内获批。一项旨在研究维奈克拉+ HMA（地西他滨、阿扎胞苷）方案有效性及安全性的Ⅰb期临床试验表明，在不适合强化疗的老年初治AML患者中，维奈克拉+ HMA方案总体有效率为67％，且耐受性良好，其中高危患者（如年龄>75岁、细胞遗传学提示预后不良、继发AML等）对该方案应答良好[9]。后续的Ⅲ期临床研究进一步证实了维奈克拉+阿扎胞苷组中位生存期（14.7个月对9.6个月）及应答率（66.4％对28.3％）均优于阿扎胞苷单药组[10]。一项比较维奈克拉+LDAC与LADC方案的多中心、双盲、随机对照的Ⅲ期临床试验结果表明，在不适合强化疗的AML患者中维奈克拉+LDAC组中位生存期（8.4个月对4.1个月）及应答率（48％对13％）显著优于LDAC单药组[11]。有研究发现携带NPM1或IDH突变的AML患者，尤其是IDH2突变对维奈克拉应答率高且能维持长期的缓解，而携带TP53突变或激酶激活性突变（如FLT3-ITD、RAS突变）的AML患者则易对维奈克拉耐药[12]。

目前维奈克拉联合HMA或LDAC是治疗老年AML的首选方案，对于不良反应的处理、药物剂量及疗程的调整是临床医师们关注的重点。

（1）不良反应的处理：治疗过程中，主要关注预防肿瘤溶解综合征（TLS），其发生的高危因素包括高WBC、NPM1突变、IDH突变、高乳酸脱氢酶、高骨髓原始细胞等。预防措施：在维奈克拉治疗开始前，建议采用羟基脲或白细胞去除术将WBC降低至25×10^9^ /L以下。从低剂量开始，每天缓慢增加剂量直至目标治疗剂量，剂量增加期间患者需要充分的静脉及口服水化碱化，同时每天监测TLS相关的实验室指标（包括钾、尿酸、磷、钙和肌酐）。第二疗程可直接予以足剂量维奈克拉口服[13]。

维奈克拉治疗过程中，几乎所有患者均会出现Ⅲ～Ⅳ级血液学不良反应，对于贫血和血小板减少处理主要是支持治疗，而对于Ⅲ～Ⅳ级白细胞减少患者，如果没有明显感染不建议使用G-CSF，建议将患者置于无菌层流床（罩）内。如果出现明显感染发热，建议行骨髓穿刺检查，如果骨髓明显抑制且原始细胞<5％，可停维奈克拉并用G-CSF。如果患者合并真菌感染，建议用棘白菌素类药物，必须用唑类抗真菌药物时则需要减少维奈克拉的剂量（见说明书）。此外，不少患者住院之前已有感染发热，应了解患者是否使用唑类抗真菌药物，如使用，起始治疗剂量要减半或减少三分之二（根据不同的唑类药物）。

（2）药物剂量调整：建议在治疗开始后第21～28天进行骨髓穿刺/活检。第21天骨髓评估提示缓解且伴有骨髓抑制的患者应停用维奈克拉，此时如果中性粒细胞明显减低可予G-CSF支持治疗，等待细胞恢复后开始下一疗程治疗。下一疗程可考虑减少维奈克拉服用天数，由28 d减至21 d或14 d，或下调33％ ～50％的HMA剂量使下一疗程周期延长至4～5周。第21天骨髓评估提示存在白血病细胞的患者继续维奈克拉治疗，第29天开始下一疗程治疗，直到下一轮第21天，再次进行骨髓评估，如骨髓缓解参照前期治疗方案停药等待，如骨髓持续未缓解并且血常规也没有改善，需考虑其他药物或临床试验。

（3）最佳疗程数：关于何时停止治疗，何为最佳疗程数目前尚无定论。维奈克拉说明书以及VIALE-A、VIALE-C两项国际Ⅲ期临床研究均建议：持续治疗直至疾病进展或不可耐受的毒性[10]–[11],[14]。2021年NCCN指南同样推荐：尽可能持续治疗，联合治疗方案的最大周期数未知，只要患者可耐受且有效，治疗可以继续进行。对于维奈克拉联合HMA或LDAC治疗，达到MRD阴性的患者，目前尚无证据支持在达到MRD阴性CR患者中停用维奈克拉联合HMA治疗，需要进一步的临床试验及更多的病例数据进行分析[15]–[16]。

2. FLT3抑制剂：吉瑞替尼（Gilteritinib）是一种新型、强效、高选择性、Ⅰ型口服FLT3/AXL抑制剂，与Ⅱ型抑制剂的不同在于吉瑞替尼通常不受激活环中突变（如D835点突变）的影响，能够结合FLT3突变的活性构象和非活性构象。

一项开放标签的全球多中心Ⅰ/Ⅱ期临床试验（CHRYSALIS试验）验证了吉瑞替尼在携带野生型或突变型FLT3基因的成人难治/复发（R/R）AML患者中的安全性、耐受性、药代动力学和药效学[17]。

此外，ADMIRAL研究[18]是一项采用充分验证的检测方法检测携带FLT3突变的R/R AML成人患者的Ⅲ期、开放标签、随机、全球多中心临床研究。在该项研究中，371例患者以2∶1的比例随机接受吉瑞替尼或挽救性化疗。吉瑞替尼组中位OS时间为9.3个月，相较于挽救性化疗（中位OS时间为5.6个月）能降低36％死亡风险，显著延长患者生存期。并且吉瑞替尼与挽救性化疗相比CR/部分血液学恢复的完全缓解（CRh）率分别为34％及15.3％。安全性方面，吉瑞替尼组≥Ⅲ级不良事件发生率低于挽救性化疗。基于ADMIRAL研究数据，吉瑞替尼相继于2018年10月、11月及2019年10月获得日本、美国FDA及欧盟EMA批准上市，并已被2020年NCCN、ESMO及CSCO指南列为治疗FLT3-ITD和FLT3-TKD突变R/R AML患者1类推荐。

3. IDH抑制剂：恩西地平（Enasidenib）是一种口服的靶向IDH2突变选择性抑制剂，为R140Q和R172K的共抑制剂，也是首个IDH突变抑制剂。于2017年8月1日被FDA批准100 mg每日1次单药治疗IDH2突变AML，主要基于一项针对IDH2突变患者的Ⅰ/Ⅱ期临床试验，其结果提示口服恩西地平使R/R AML患者CR率达19.3％，有效率高达40.3％，首次缓解中位时间1.9个月,中位OS时间为9.3个月，1年OS率为39％，达到CR和PR的患者有更好的长期生存，少部分患者生存达3～4年[19]。

IDH1抑制剂艾伏尼布（Ivosidenib）的临床试验结果一样令人鼓舞，DiNardo等[20]报道了一项多中心、开放标签、剂量递增和剂量扩展Ⅰ/Ⅱ期试验的结果，在主要疗效人群（125例患者）中，CR或CRh率为30.4％，中位客观有效时间为8.2个月，中位生存时间为8.8个月。FDA于2018年7月20日批准了艾伏尼布500 mg每日1次用于伴IDH1突变的R/R AML患者的治疗。基于一项艾伏尼布联合阿扎胞苷治疗IDH1突变且不适合强化化疗的AML的Ⅰb期试验[21]，2019年5月2日FDA批准了艾伏尼布作为年满75岁或患有合并症而不能使用强烈诱导化疗的AML患者的一线治疗用药。

4. Glasdegib：Glasdegib是一种新型口服smoothened（SMO）抑制剂，可调节hedgehog信号通路的活性以起到白血病治疗作用。在Cortes等[22]报道的一项Ⅰb/Ⅱ期随机临床试验中，患者接受LDAC或联合Glasdegib 100 mg/d治疗。88例患者接受LDAC联合Glasdegib治疗，44例患者仅接受LDAC治疗。联合Glasdegib后显著延长了生存时间，中位OS时间分别为8.8个月和4.9个月，1年OS率分别为59.8％和38.2％。Glasdegib联合组的总体缓解率（包括CR、CRi和MLFS）为26.9％，而单用LDAC组的总体缓解率为5.3％，其中CR率分别为17.0％和2.3％。2018年11月21日美国FDA批准Glasdegib联合LDAC用于年满75岁或不适合强化化疗的AML患者的一线治疗。

5. Magrolimab：抗CD47单抗Magrolimab是一种新型的免疫疗法和靶向LSC制剂，可阻断关键的巨噬细胞检查点。在2020年美国血液学年会上，Magrolimab+阿扎胞苷方案治疗初治AML（包括TP53突变的AML）患者最新的Ⅰb期研究，共34例AML患者可进行疗效评估，总体客观缓解率为65％（44％ CR，12％ CRi，3％ PR，6％ MLFS），相比于阿扎胞苷单药组，使用Magrolimab+阿扎胞苷方案组获缓解更快。TP53突变患者中，客观缓解率71％（42％ CR，19％ CRi，5％ MLFS），TP53野生型和突变型患者中位OS时间为18.9个月和12.9个月[23]。未来该药物在AML的治疗中也有广阔的前景。

四、展望

老年AML的治疗仍是血液科临床医师的重大挑战。目前靶向干预手段日益增多，临床医师应根据老年AML的分子特征和个体差异，合理选择运用靶向药物，包括HMA，充分发挥AML靶向治疗的精准、高效、低毒性。
